# Assisted ventilation withdrawal in motor neuron disease: updated results

**DOI:** 10.1136/spcare-2025-005389

**Published:** 2025-03-24

**Authors:** Lucy Bleazard, Jonathan Palmer, David Wenzel, Thomas Jeffery, Christina Faull

**Affiliations:** 1 Population Health Sciences, University of Leicester, Leicester, UK; 2 LOROS Hospice, Leicester, UK; 3 University Hospitals Plymouth NHS Trust, Plymouth, UK

**Keywords:** Education and training, Home Care, Hospice care, Palliative Care, Symptoms and symptom management, Terminal care

## Abstract

**Introduction:**

Patients with ventilator-dependent motor neuron disease (MND) may request withdrawal of their assisted ventilation. Facilitating this process as a healthcare professional (HCP) can be emotionally and practically challenging. The Association for Palliative Medicine (APM) issued guidance to support HCPs and invited anonymised accounts of the withdrawal process to provide an update on the guidance.

**Methods:**

HCPs submitted anonymised accounts via email. Quantitative data was analysed descriptively in Excel. Free-text comments were analysed thematically using an inductive, iterative approach.

**Results:**

68 HCPs submitted 95 accounts of ventilation withdrawal between 2015 and 2024. Most patients received medications pre-withdrawal (94%), primarily a combination of an opioid and midazolam, mostly subcutaneously. Younger patients tended to need higher doses to achieve adequate symptom management prior to withdrawal. Practices of weaning the ventilator varied significantly between respondents. The median time to death following withdrawal of ventilation was 30 min, with three-quarters of patients dying within 2 hours.

**Conclusion:**

This is the largest data set to date regarding the withdrawal of assisted ventilation in MND. This updated analysis reaffirms that a personalised, titrated approach remains appropriate and effective. The revised APM Guidance 2025 incorporates new sections on recommendations for managing the ventilator.

**WHAT IS ALREADY KNOWN ON THIS TOPIC**

- Withdrawing assisted ventilation at the request of a patient with MND can be complex and challenging.
- Support for HCPs is available through the APM guidance.

**WHAT THIS STUDY ADDS**

- The personalised, titrated approach advocated by the APM guidance remains appropriate.
- Challenges are identified, including predicting time to death and ventilator management.

**HOW THIS STUDY MIGHT AFFECT RESEARCH, PRACTICE OR POLICY**

- An updated version of the APM guidance will be released in 2025.

## Introduction

 Patients with ventilator-dependent motor neuron disease (MND; or amyotrophic lateral sclerosis) may request that their assisted ventilation is stopped if the benefits no longer outweigh the burdens. The withdrawal process encompasses the professional responsibility to produce an individualised management plan including minimisation of distress, breathlessness and agitation.^1^ Healthcare professionals (HCPs) have said that facilitating this process can be both practically and emotionally challenging.^2 3^

In response, the Association for Palliative Medicine (APM) published widely endorsed guidance to support HCPs working with patients who have requested withdrawal of their assisted ventilation.^4^ HCPs using the guidance were asked to submit anonymised accounts of their experience to further inform best practice recommendations.

A prospective evaluation of the first 46 responses was published in 2020.^5^ This paper provides an update of the findings from almost 100 responses received between 2015 and 2024, providing additional insight into the withdrawal process from practitioners across the UK and informing an evidence-based update of the guidance.

## Methods

After withdrawing assisted ventilation at the request of a patient with MND, an anonymised proforma was completed by a HCP providing the following information:

- Patient demographic and clinically contextual information
- Information about the HCPs and their involvement
- Medications used before, during and after withdrawal of ventilation
- Duration of the process
- Any challenges in managing symptoms
- Assessment of the experience of the family
- Personal reflection

Anonymised accounts were submitted via email and entered into Excel for analysis. Quantitative data were analysed descriptively. Free-text comments were analysed thematically using an inductive, iterative approach.^6^ The text was manually coded and themes were collaboratively developed by three clinicians.

## Results

Data pertaining to 95 patients was submitted by 68 HCPs, including 56 palliative medicine doctors, 9 nurses and 3 allied health professionals. The majority of patients were male (75%), used non-invasive ventilation as opposed to invasive ventilation (94%), were dependent on their ventilator for >22 hours per day (92%), could only manage for a maximum of a few minutes off their ventilator (82%), were fully alert at the time of withdrawal (80%) and directly asked for ventilation to be stopped (88%) (i.e. not a best interests decision or result of an Advance Directive to Refuse Treatment). Almost half of withdrawals took place in patients’ homes (48%), half at inpatient hospice units (48%), and a small number in care homes and acute hospitals (4%).

### Symptom management during the withdrawal process

The APM guidance advocates an individualised, titrated approach to symptom management, tailored to the patient’s ventilator dependence, symptom burden when ventilation is interrupted, drug history, and the patient and family’s preferences.

Table 1 demonstrates the median and mode doses of morphine-equivalent and midazolam required by patients both before and after ventilation was withdrawn, as well as the total doses used throughout the process. It was observed that younger patients (<50 years old) tended to require larger doses of medication to achieve adequate sedation prior to withdrawing the ventilation, although they represented the smallest age category in the sample.

**Table 1:** Medication doses before, after withdrawal and in total use by age group

	Dosage	Age 30–50	Age 51–70	Age>70
**Before withdrawal**	**Midazolam**Median (mg)Mode (mg)Range (mg)	n=11303010–120	n=4115105–55	n=2715102–45
	Opioid*Median (mg)Mode (mg)Range (mg)	n=1125405–45	n=391555–55	n=251552–47.5
**After withdrawal**	**Midazolam**Median (mg)Mode (mg)Range (mg)	n=6101010–20	n=2610105–30	n=176.5100.5–30
	Opioid*Median (mg)Mode (mg)Range (mg)	n=610105–30	n=211055–40	n=12752–20
**Total**	**Midazolam**Median (mg)Mode (mg)Range (mg)	n=11404010–120	n=49201010–60	n=2820200.5–55
	Opioid*Median (mg)Mode (mg)Range (mg)	n=1130307.5–60	n=4720107.5–60	n=252054–47.5

* Opioid doses given as subcutaneous morphine equivalent.

Most respondents used the subcutaneous route for parenteral medication administration (82%), however, some preferred intravenous (16%) as this allowed for more rapid onset and responsive titration. 94% of patients received at least one dose of medication prior to stopping the ventilation (mode=1 dose, range=1–8 doses), usually a combination of opioid and midazolam. 56% of patients required medication after the ventilation was withdrawn. Around 10% of patients required the addition of levomepromazine as a sedative, and one patient required the use of phenobarbital.

### Management of the ventilator

Almost half of respondents (49%) chose to gradually reduce the ventilator settings (wean) prior to withdrawal. Mean ventilator pressures were IPAP (inspiratory positive airway pressure) 19.6 cmH2O and EPAP (expiratory positive airway pressure) 6.0 cmH2O. The approach to how ventilator settings were adjusted varied significantly. Some respondents focused solely on reducing back-up respiratory rate, while others paired this with an incremental reduction in pressure. The stepwise reduction in pressures also varied; some cautiously reduced pressures by 2–3 cmH2O at a time, whereas others halved the pressure and then withdrew the ventilation if no distress was observed.

Patients’ specialist home ventilation teams were not always able to be present at the time of withdrawal. This occasionally coincided with difficulties operating the ventilator due to unfamiliarity with the machine, with some respondents reporting being unable to unlock the device, adjust the settings or manage the alarms.

Almost all patients (97%) chose to have the ventilator mask or interface removed before death. This was usually done by an HCP, but in 19% of cases, it was performed by the patient’s family.

### Duration of the withdrawal process

The median time from the decision to actively start the withdrawal process to removing or switching off the ventilator (or ‘time to withdrawal’—TTW) was 46.5 min, and the median time from that withdrawal to death of the patient (or ‘time to death’—TTD) was 30 min. 74% of patients had a TTD of<2 hours. All patients receiving invasive ventilation died within less than 30 min of ventilation being stopped. The median total time of the withdrawal process to death of the patients (TTW+TTD) was 105 min.

There remains a significant variation in the duration of the withdrawal process between patients, particularly in the TTD, which ranged from 10 min to over 54 hours. Notably, for 10% of patients, this was longer than a working day. There was a distinct lack of predictability as to which patients would live for several hours after ventilation was stopped.

### Professionals’ reflections on the withdrawal process

HCPs mostly perceived the withdrawal process as positive for the patient and their family (n=88%), with only five patients experiencing particularly challenging symptoms to manage, all commented on in the 2020 analysis.

When reflecting on their own experience, 67% of HCPs felt it was positive, with many finding it a smooth and rewarding process facilitated by the APM guidance. However, many felt there were opportunities for improvement with 43% stating things they would do differently in future. These were categorised into the following themes:

#### Practical and technical considerations

Many participants commented on practical aspects of the withdrawal they would consider, including turning off air mattresses, remembering to bring a stethoscope and ensuring knowledge of unlocking and altering pressure settings on the ventilator.

#### Early familiarisation with patient, family and wider care teams

I only met the patient on the day … it would have been less stressful for me to see the patient and the setting prior to the withdrawal.

Arranging meetings with the patient, their carers and loved ones, and their community teams (eg, home ventilation) as part of the planning process was often recommended. The opportunity to discuss ethical and legal considerations of withdrawing life-sustaining treatment within the wider multidisciplinary team was also felt to be important.

#### Planning for a longer time to death than may be expected

It’s easier to cancel support staff than try to find it if needed.

Although most patients died within 2 hours of their ventilation being withdrawn, some respondents wished they had planned for the possibility of a longer withdrawal, particularly when this stretched beyond the working day. Some recommended that this is discussed with the patient and their loved ones prior to starting the withdrawal to minimise the family’s distress if the process unexpectedly lasts longer than anticipated.

## Discussion

This is the largest data set reported to date regarding the withdrawal of assisted ventilation for patients with MND. The updated analysis reaffirms that the personalised, titrated approach advocated by the APM guidance remains appropriate and effective. Younger patients appear to potentially need higher doses of medication to achieve good symptom management; however, this should always be considered on an individual basis. Reflections from HCPs reveal the rewarding yet challenging nature of the withdrawal process and the value of the APM guidance.

The findings highlight that management of the ventilator should be prepared for and discussed with the patient’s home ventilation team if possible, including inviting members of the home ventilation team to participate in the withdrawal if not already involved. Overlooking practical considerations, such as knowing how to unlock and alter the ventilator settings, can lead to unnecessary delays and anxiety for all involved. In response, the revised APM Guidance 2025 incorporates a section on recommendations for managing the ventilator, including instructions on weaning respiratory pressures.

Open and proactive communication with all members of the patient’s wider caregiving team is recommended, as long as this is achieved in a timely manner so as not to unduly delay the process of achieving the patient’s wishes. Transparency with the patient’s loved ones about the practicalities involved and the small possibility of a longer time to death even when very ventilator-dependent should be considered.

## Conclusion

This study provides new information for HCPs to discuss and prepare for ventilation withdrawals, particularly with regards to ventilator management. The findings support the ongoing use of the APM guidance and have informed an evidence-based update to be released in early 2025.

## References

[1] Lords Hansard (1997). House of lords debate re: annie lindsell.

[2] Faull C, Rowe Haynes C, Oliver D (2014). Issues for palliative medicine doctors surrounding the withdrawal of non-invasive ventilation at the request of a patient with motor neurone disease: a scoping study. BMJ Support Palliat Care.

[3] Phelps K, Regen E, Oliver D (2017). Withdrawal of ventilation at the patient’s request in MND: a retrospective exploration of the ethical and legal issues that have arisen for doctors in the UK. BMJ Support Palliat Care.

[4] Association for Palliative Medicine (2015). Withdrawal of Assisted Ventilation at the Request of a Patient with Motor Neurone Disease Guidance for Professionals.

[5] Faull C, Wenzel D (2022). Mechanical ventilation withdrawal in motor neuron disease: an evaluation of practice. *BMJ Support Palliat Care*.

[6] Braun V, Clarke V (2006). Using thematic analysis in psychology. Qual Res Psychol.

